# Effectiveness, Healthcare Resource Utilization and Adherence to Subcutaneous Interferon Beta-1a According to Age in Patients With Multiple Sclerosis: A Cohort Study Using a US Claims Database

**DOI:** 10.3389/fneur.2021.676585

**Published:** 2021-07-22

**Authors:** Arthur Allignol, Emmanuelle Boutmy, Meritxell Sabidó Espin, Kurt Marhardt, Patrick Vermersch

**Affiliations:** ^1^Merck Healthcare KGaA, Darmstadt, Germany; ^2^Merck Gesellschaft mbH (an affiliate of Merck KGaA), Vienna, Austria; ^3^Univ. Lille, Inserm U1172 LilNCog, CHU Lille, FHU Precise, Lille, France

**Keywords:** adherence, age, healthcare utilization, subcutaneous interferon beta-1a, multiple sclerosis, relapse

## Abstract

**Background:** It is thought that older patients with multiple sclerosis (MS) may present with a different clinical disease phenotype, and therefore respond to subcutaneous interferon beta-1a (sc IFN β-1a) differently to younger patients. However, few real-world data are available concerning the effectiveness of sc IFN β-1a according to age. Using data from US claims databases, this cohort analysis aimed to determine the differences in relapse rates, healthcare utilization, treatment adherence, and discontinuation according to pre-defined age groups.

**Methods:** Patient data were pooled from the IBM® MarketScan® Commercial Claims Database and Medicare Supplemental Database. Patients with a confirmed MS diagnosis who initiated treatment with sc IFN β-1a between July 01, 2010 and December 31, 2015, along with at least 6 months continuous enrolment in a healthcare plan, were followed from first prescription (index date) until date of discontinuation, treatment switch, or end of observation period (1 year after index date).

**Results:** Of the 5,340 patients included in the analysis, there was a high proportion of patients free from relapse across all age groups (range: 94.1–95.4%), with a numerical decrease in the number of MRI performed by age (mean: 0.25, 18–30 years; 0.20, 31–40 years; 0.16, 41–50 years; 0.14, ≥51 years). Adherence (≥80%) was seen to increase with age (77.6%, 18–30 years; 79.6%, 31–40 years; 81.3%, 41–50 years; 84.0%, ≥51 years), at the same time as a non-significant decrease in discontinuation (incidence rate: 79.91, 73.01, 71.75, 68.71%).

**Conclusion:** The effectiveness of sc IFN β-1a does not appear reduced as a consequence of age in this real-world setting. Older patients had lower discontinuation rates and reduced disease activity, reflected in lower relapse rates and fewer MRI scans compared with younger patients.

## Introduction

Multiple sclerosis (MS) is a chronic inflammatory disease affecting over 900,000 people in the United States (US) alone, with a prevalence of 288 per 100,000 of the population (1, 2). The incidence of MS typically increases after the age of 18 years, with most patients diagnosed between the ages of 20–40 years (3). Several clinical studies have suggested that the age of onset influences the disease course of MS, but there is limited consistency in these findings (4–6).

Patients with MS may experience disability progression at varying rates as a consequence of the disease etiology (7), with the most frequent form of MS, relapsing-remitting, showing an age-dependent transition to a progressive disease course (8). Disability is predominant in progressive forms of MS, and as older patients may present with progressive MS, it is likely they may have a greater disability accumulation compared with younger patients and therefore may not respond to disease-modifying therapies (DMTs) in the same manner as their younger counterparts (9).

Interferons have been a standard first-line treatment for patients with MS since the 1990s, with subcutaneous interferon beta-1a (Rebif®, herein referred to as sc IFN β-1a) providing an estimated cumulative exposure of 1,809,458 patient-years across more than 100 countries worldwide (up to January 08, 2021). Previous clinical trials have shown that sc IFN β-1a maintains some effectiveness with increasing age (10, 11), whereas other DMTs have shown a decrease in effectiveness over time (12). There is, however, a need to increase the real-world evidence pool concerning the effectiveness of sc IFN β-1a across different age groups. One such source of data on sc IFN β-1a is found within large healthcare insurance databases.

Using US claims databases, this study therefore set out to assess whether sc IFN β-1a-treated patients with MS have differences in baseline characteristics, relapse rates, healthcare utilization rates, and treatment adherence and discontinuation according to pre-defined age groups.

## Methods

### Data Source

This non-interventional cohort study involved an analysis of secondary data collected from the IBM® MarketScan® Commercial Claims Database and Medicare Supplemental Database (hereafter called the MarketScan® databases). The MarketScan® databases contain de-identified administrative claims and eligibility records of over 125 million commercially insured individuals from all US regions, including patients who have Medicare Supplemental Insurance paid for by their employers. Claims for in- and out-patient medical services, pharmacy-related claims and patient demographics, including age, sex, and region, are recorded within the databases.

### Patient Selection and Study Design

**Figure 1:**
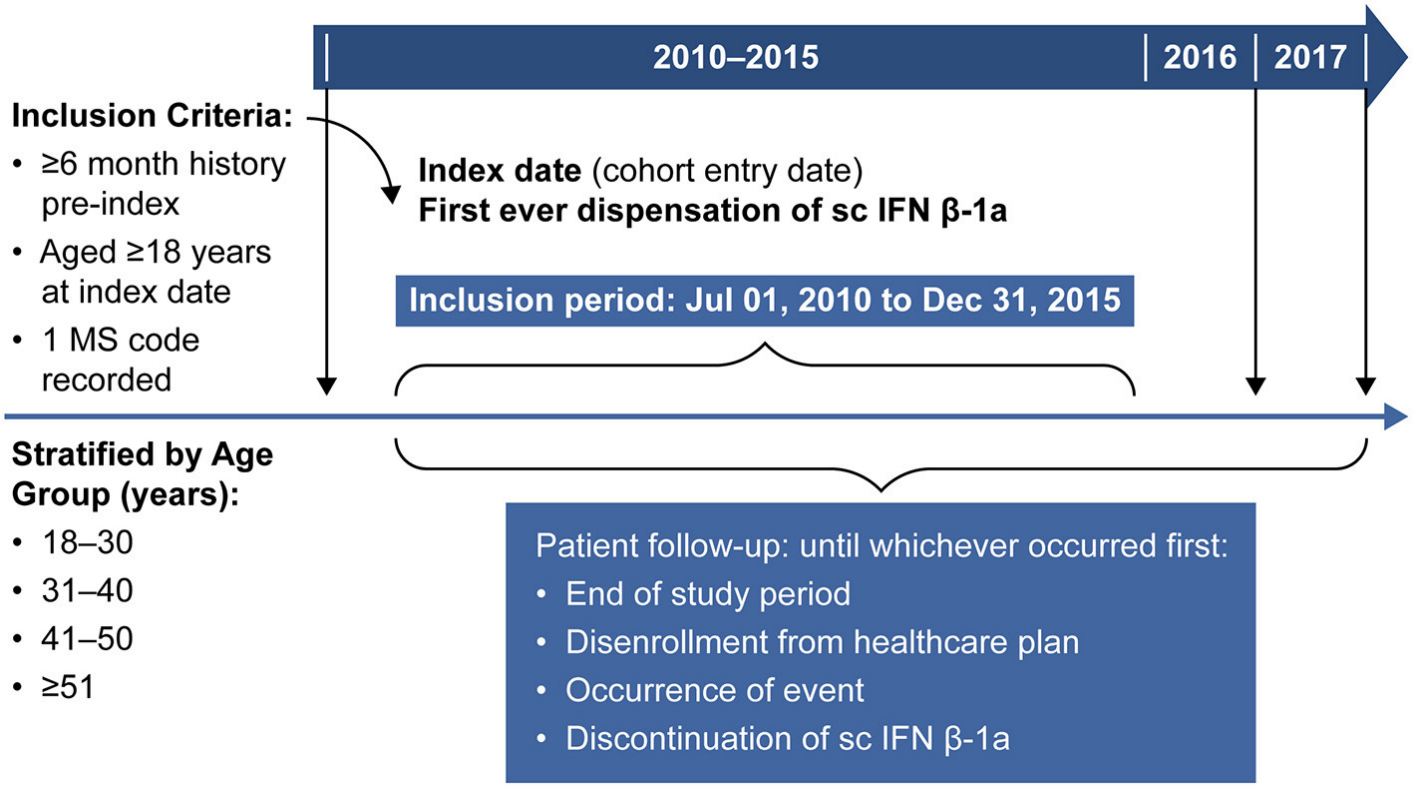
Study design. MS, multiple sclerosis; sc IFN β-1a; subcutaneous interferon beta-1a.

The study period included patients recorded in the MarketScan® databases between January 01, 2010 and December 31, 2017. Data for patients with MS who had initiated treatment with sc IFN β-1a between July 01, 2010 and December 31, 2015, regardless of having prior treatment with other DMTs, were eligible for inclusion in the study (Figure 1). In addition, data were only included if patients were aged ≥ 18 years in the year of the index date (defined as the date of first dispensation of sc IFN β-1a), had a diagnosis of MS based on the presence of ≥1 medical claim with a primary or secondary code for MS [International Classification of Diseases, 9th revision, Clinical Modification (ICD-9-CM: 340) or G35 in ICD-10], had ≥6 months of medical history prior to index date, operationalized as being continuously enrolled in a healthcare plan in the 6 months prior to index date (allowing for 30-day gaps). No exclusion criteria were included in the study design.

### Covariates and Outcomes

Baseline covariates, including MS disease variables and medication history, were assessed during the 6-month pre-index period. The follow-up period for all patients was from the index date until the end of the study, or disenrollment from the healthcare insurance plan, occurrence of an event, or discontinuation of sc IFN β-1a therapy (whichever occurred first).

The primary objective of the study was to compare the proportion of patients free of relapses 1 year after treatment initiation across all age groups. Relapse episodes were identified based on a published claims-based algorithm published by Marriott et al., which has a sensitivity of 70% and a specificity of 100% (13). Further information on this claims-based algorithm is provided in the Supplementary Material. Secondary objectives were to compare the time of first relapse after treatment initiation, annualized relapse rates (ARR), healthcare resource utilization [HRU, defined as the quantification of the use of medical services by an individual over a period of time, and are herein reported on a per-patient-per-month (PPPM) basis], and treatment adherence and discontinuation at 1 year after the initiation of sc IFN β-1a across all age groups. For patients who discontinued sc IFN β-1a treatment, the subsequent DMT(s) were recorded. Patient co-morbidities and comedications were also assessed at 1 year after treatment initiation.

Treatment adherence was defined using a medication possession ratio (MPR), taking into consideration the number of days of dispensed medication within a refill interval and the number of days within the refill interval. The MPR was reported as a continuous variable and percentage using the conventional cut-off values of <80% to define non-adherent patients, 80–120% for adherent patients, and >120% for over-compliant patients, who obtained their medication up to 10 days in advance thus causing an increase in the number of days of dispensed medication while the days within the refill interval remain unchanged (14).

### Statistical Analysis

All categorical variables were summarized as frequencies, percentages, and number of missing values. Continuous variables were summarized using mean, standard deviation (SD), median, interquartile range (IQR), minimum, and maximum values.

This analysis focuses on the data from the first year after treatment initiation, with all study outcomes described and compared by age groups (18–30, 31–40, 41–50, and ≥51 years). For all analyses, except for the outcome of discontinuation, patients' follow-up was stopped at index treatment discontinuation.

The probability of being free from relapse was estimated using the Cumulative Incidence function. Sub-distribution hazard ratios and 95% confidence intervals (CI) were calculated using a Fine and Gray model (Equation 1) to quantify the effect of age on time to first relapse while considering discontinuation as competing risks.

$$\begin{array}{l} \Upsilon_{k}\left(t;z\right)=\Upsilon_{k0}\left(t\right)\exp\left(z^{\prime }\beta \right) \end{array}$$

The left-hand side of Equation 1 shows the sub-distribution hazard for cause “k” (i.e., relapses occurring at 1 year) assuming proportional hazards on the sub-distribution hazard. The time at risk of relapse was considered to begin at the date of sc IFN β-1a exposure (the index date). ARRs were calculated as the ratio between the number of relapses defined in a given period, and the number of person-years.

HRU was reported on a PPPM basis over 1 year and according to age groups. For each patient, monthly HRU was calculated by dividing the overall HRU by the total sc IFN β-1a exposure period, allowing for a maximum of 12 months (Equation 2).

$$\begin{array}{l} HRU_{PPPM}=\;\frac{HR\;uses\;within\;T}{Number\;of\;months\;of{\;T}^{\prime }\;} \end{array}$$

Where T (time period) was equal to time from the index date to the end of sc IFN β-1a exposure.

The proportion of patients free of discontinuation at 1 year were analyzed using the Kaplan-Meier estimator, while differences in time to discontinuation between age groups were assessed using a Cox proportional hazards model (Equation 3), in which the censoring event was a follow-up of more than 1 year, or other following events occurring within the first year after index date: loss of insurance coverage for more than 40 days (loss of follow-up), or the end of the study period (December 31, 2017).

$$\begin{array}{l} \alpha \left(t\right)=\;\alpha_{0}\left(t\right)\;\exp\left(z^{\prime }\beta \right) \end{array}$$

Because age as a factor cannot be modified, adjusting for confounders via a propensity score would be conceptually difficult. Direct regression would also not allow for adjustment of many variables due to the low number of relapse events. Thus, all comparisons presented in this study are unadjusted.

Two sensitivity analyses were conducted for the primary outcome. The first considered a grace period of 90 days after discontinuation to account for further relapse events after the last prescription. The second sensitivity analysis considered a baseline period of 12 months.

### Ethics

This study involves an analysis of secondary data in which all records are anonymized to protect the privacy of patients and providers. In the MarketScan® databases, patient confidentiality is maintained through compliance with the Health Insurance Portability and Accountability Act (HIPAA) regulations of 1996. Accordingly, ethics committee and institutional review board approval were not required for the study.

## Results

### Patients

**Figure 2:**
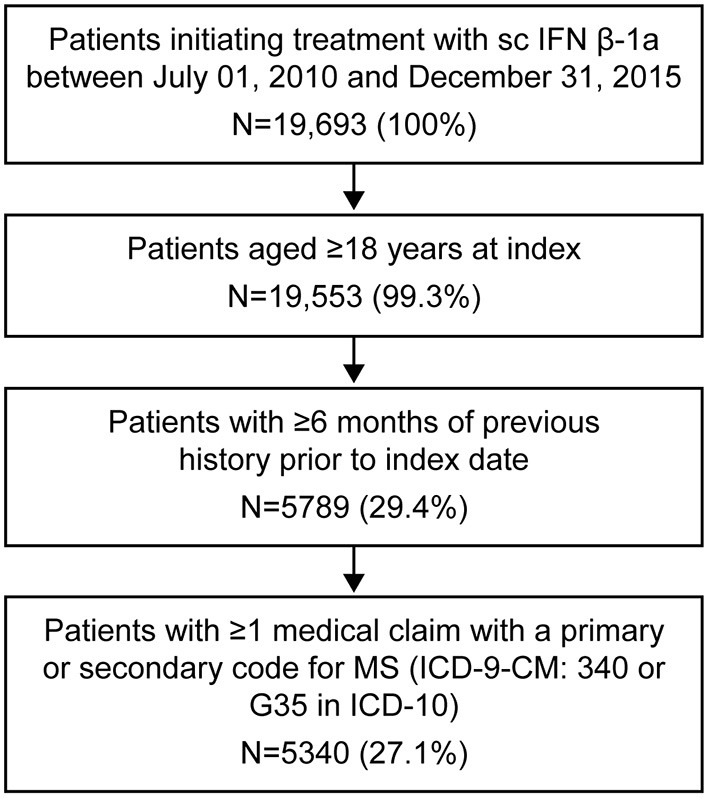
Patient flow selection. ICD, International Classification of Diseases, Revision 9; MS, multiple sclerosis; sc IFN β-1a, subcutaneous interferon beta-1a.

Overall, the MarketScan® databases held data for 19,693 patients who initiated treatment with sc IFN β-1a between July 01, 2010 and December 31, 2015. Of these patients, 5,340 (27.1%) met all of the inclusion criteria (Figure 2).

Patient demographics and characteristics are shown in Table 1. Within the study population, the majority of participants, across all age groups, were female (range: 74.5–78.2%) and mostly treatment-naïve (range: 64.5–75.7%). Of patients aged 18–30 years, 154 (19.9%) had ≥1 relapse during the baseline period compared with 139 (9.5%) patients aged ≥51 years.

**Table 1:** Patient demographic and clinical characteristics at index.

	**Age group, years**			
**Baseline variables**	**18–30(*N* = 773)**	**31–40(*N* = 1,468)**	**41–50(*N* = 1,630)**	**≥51 (*N* = 1,469)**
Age, years, mean (SD)	25.82 (3.24)	35.83 (2.82)	45.50 (2.84)	57.27 (4.99)
Min–Max	18–30	31–40	41–50	51–82
Sex, female	576 (74.5)	1,118 (76.2)	1,247 (76.5)	1,148 (78.2)
**Prior DMT use^a^**				
Naïve	585 (75.7)	1,073 (73.1)	1,136 (69.7)	947 (64.5)
Use of IM	162 (21.0)	333 (22.7)	422 (25.9)	430 (29.3)
Use of IS	30 (3.9)	66 (4.5)	85 (5.2)	113 (7.7)
Use of IM and IS	1 (0.1)	1 (0.1)	4 (0.3)	9 (0.6)
Glucocorticoid use^b^	406 (52.5)	650 (44.3)	702 (43.1)	549 (37.4)
Relapses during baseline, mean (SD)	0.21 (0.44)	0.15 (0.38)	0.14 (0.38)	0.10 (0.31)
Min–Max	0–3	0–2	0–3	0–2
Number of patients with ≥1 relapse during baseline	154 (19.9)	216 (14.7)	204 (12.5)	139 (9.5)

*Data presented as n (%) unless otherwise stated*.

a *DMTs included immunomodulators: Avonex® (IFN β-1a), Betaferon® (IFN β-1b), Copaxone® (glatiramer acetate), Extavia® (IFN β-1b), Glatopa™ (glatiramer acetate, generic equivalent of Copaxone 20 mg), Plegridy® (pegylated IFN β-1a), Rebif® (IFN β-1a); and immunosuppressants: Aubagio® (teriflunomide), Gilenya® (fingolimod), Tecfidera® (dimethyl fumarate), Lemtrada® (alemtuzumab), Tysabri® (natalizumab), methotrexate, mitoxantrone, cyclophosphamide, mycophenolate mofetil, Imuran® (azathioprine), rituximab, and tacrolimus*.

b *Glucocorticoids included: betamethasone, cortisone, dexamethasone, fludrocortisone, hydrocortisone, methylprednisolone, prednisolone, and triamcinolone*.

*COPD, chronic obstructive pulmonary disease; DMT, disease-modifying therapy; IFN, interferon; IM, immunomodulator; IQR, interquartile range; IS, immunosuppressant; SD, standard deviation*.

The frequencies of co-morbidities and concomitant medication use for all patient groups are reported in Supplementary Table 1. Generally, patients aged ≥51 years reported a higher proportion of co-morbidities than younger patients. The most common co-morbidity in this study population was hypertension, which was reported in 29.3% of patients aged ≥51 years compared with 3.2% of patients aged 18–30 years. The older age group also reported a higher proportion of co-medications for conditions such as bladder dysfunction, spasticity, emotionalism, depression, and pain treatments (all of which were reported in >20% of patients aged ≥ 51 years).

Overall, 1,713 patients switched treatments after discontinuation of sc IFN β-1a (Supplementary Table 2). Among these patients, the most frequent switch was to dimethyl fumarate (Tecfidera®), a trend that was observed across all age groups.

### Relapses and Annualized Relapse Rate

**Figure 3:**
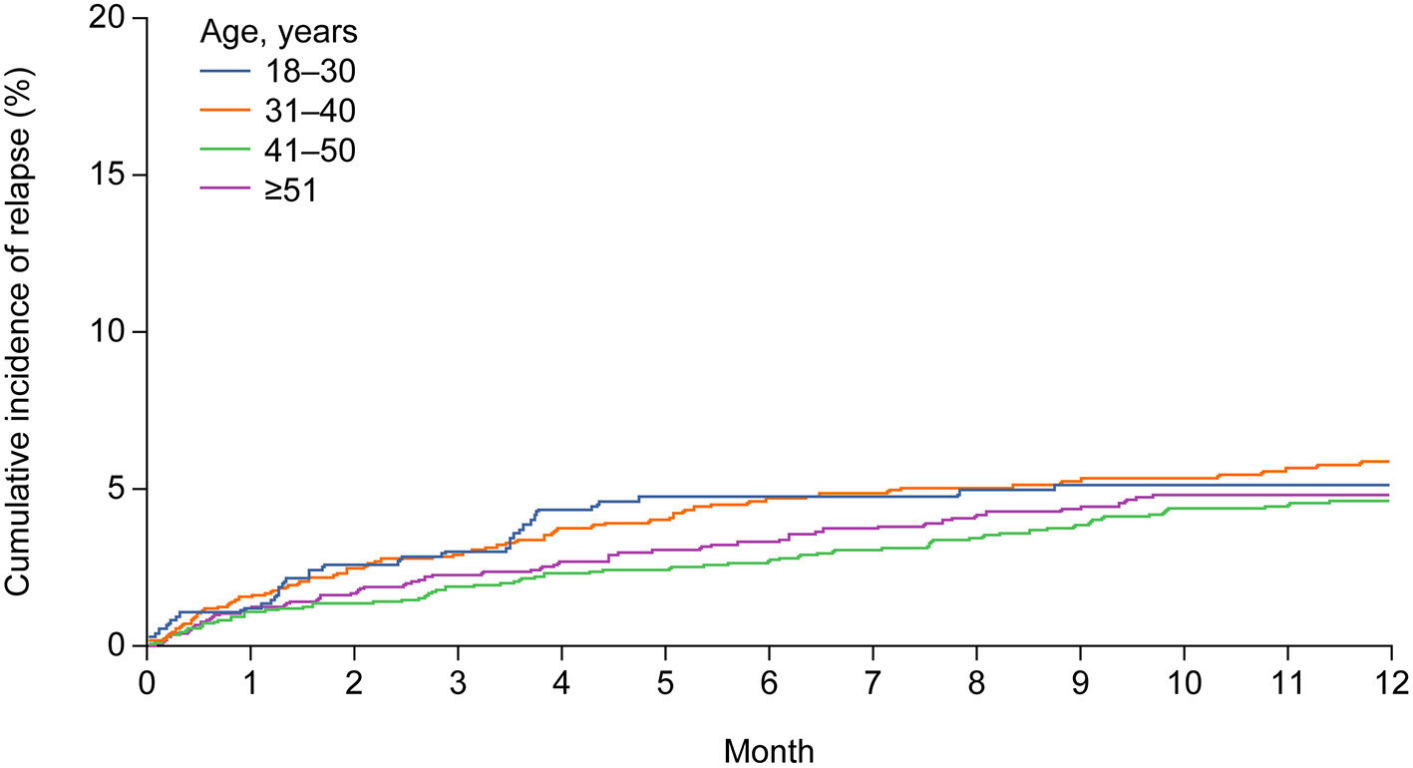
Cumulative incidence of relapse during 1 year of follow-up, according to age group.

As previously mentioned, the number of relapses in the baseline period decreased with increasing age. At 1 year after initiating treatment with sc IFN β-1a, the proportion of patients free from relapse was high across all age groups (range: 94.1–95.4%, Table 2), with a similar cumulative incidence of relapse (Figure 3). Results from the sensitivity analyses were also consistent with these findings (Supplementary Table 3).

**Table 2:** Proportion of patients relapse free and annualized relapse rate at 1 year, and time to first relapse after initiation of sc IFN β-1a treatment.

	**Age group, years**			
	**18–30^a^(*N* = 773)**	**31–40(*N* = 1,468)**	**41–50(*N* = 1,630)**	**≥51 (*N* = 1,469)**
**Relapse free at 1 year**				
Proportion of patients (95% CI)	94.87 (93.24–96.51)	94.14 (92.83–95.44)	95.39 (94.28–96.50)	95.19 (94.01–96.36)
Unadjusted SHR (95% CI)	–	1.09 (0.73–1.62)	0.82 (0.55–1.24)	0.88 (0.58–1.33)
*p*-value	–	0.67	0.34	0.55
**Time to first relapse**				
Proportion of patients^b^ (95% CI)	89.73 (86.56, 92.90)	89.56 (86.31–92.81)	89.41 (86.75–92.07)	90.35 (87.87–92.82)
Unadjusted SHR (95% CI)	–	0.98 (0.70–1.38)	0.85 (0.60–1.19)	0.87 (0.62–1.24)
*p-*value	–	0.91	0.34	0.45
**ARR at 1 year**				
Number of events	41	80	72	71
Person-years	417.97	821.84	937.95	848.48
ARR (95% CI)	0.10 (0.07–0.13)	0.10 (0.08–0.12)	0.08 (0.06–0.10)	0.08 (0.07–0.11)
ARR ratio (95% CI)	–	1.00 (0.68–1.45)	0.80 (0.53–1.15)	0.85 (0.58–1.25)

a *Reference group*.

b *Proportion of patients without a relapse in their follow-up period*.

*ARR, annualized relapse rate; CI, confidence interval; sc IFN β-1a; subcutaneous interferon beta-1a; SHR, subdistribution hazard ratio estimates*.

Across all age groups, a high proportion of patients did not have a first relapse during the follow-up period, ranging from 89.4 to 90.4% (Table 2). The median (IQR) duration of follow-up was 5.98 (2.10–13.40), 6.41 (2.53–15.41), 7.23 (2.33–18.32), and 7.13 (2.33–17.84) months in the 18–30, 31–40, 41–50, and ≥51 years' age groups, respectively. The ARR at 1 year after treatment initiation decreased numerically from 0.10 (95% CI 0.07–0.13) in patients 18–30 years to 0.08 (95% CI 0.07–0.11) in patients aged ≥51 years, although the differences were not statistically significant (Table 2).

### Healthcare Resource Utilization

**Figure 4:**
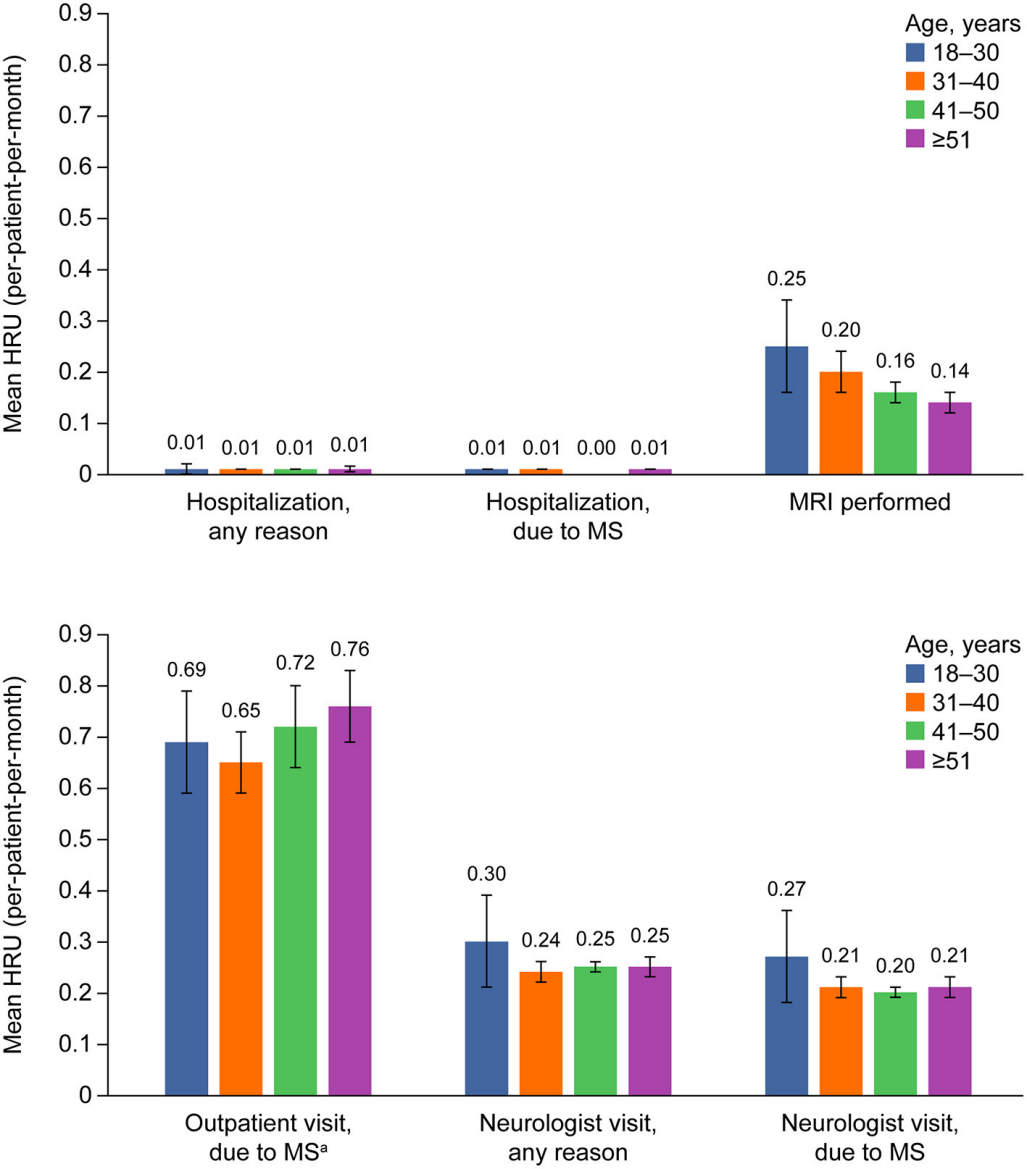
Health care utilization over 1 year after treatment initiation, according to age group. Error bars show the 95% CIs for each outcome by age. ^a^Out-patient visits include: visits with neurologist, general practitioner, and nurse; hospital visits, and emergency room visits. Excluded: visits with psychologist, psychiatrist, speech therapist, and rehabilitation. HRU, healthcare resource utilization; MRI, magnetic resonance imaging; MS, multiple sclerosis.

During the first year after treatment initiation, the mean number of hospitalizations for any reason, as well as those due to MS, were low across all age groups (all ≤ 0.01 visits) (Figure 4).

The mean number of MRIs performed numerically decreased by age, from 0.25 (95% CI 0.16–0.34) in patients aged 18–30 years, to 0.14 (95% CI 0.12–0.16) in patients aged ≥ 51 years. However, there was a numerical increase in the mean number of outpatient visits due to MS in older patients; 0.69 (95% CI 0.58–0.79) in patients aged 18–30 years vs. 0.76 (95% CI 0.69–0.84) in patients aged ≥ 51 years. The differences observed across age groups were not statistically significant.

The mean number of neurologist visits for MS was also highest among younger patients, with a mean number of visits per month equal to 0.27 (95% CI 0.18–0.36) for patients aged 18–30 years compared with 0.21 (95% CI 0.19–0.23) visits for patients aged ≥ 51 years.

### Treatment Adherence

Over the follow-up period, the proportion of patients considered adherent to treatment with sc IFN β-1a ranged from 77.6% in patients aged 18–30 years to 84.0% in patients aged ≥51 years (Table 3). The mean ± standard deviation (SD) MPR also increased by age, from 87.6 ± 12.80% in patients aged 18–30 years to 89.5 ± 12.48% in patients aged ≥ 51 years. Rates of discontinuation showed the opposite trend although no statistically significant differences were apparent (Table 3).

**Table 3:** Treatment adherence and discontinuation at 1 year after treatment initiation.

	**Age group, years**			
	**18–30^a^(*N* = 773)**	**31–40(*N* = 1,468)**	**41–50(*N* = 1,630)**	**≥51 (*N* = 1,469)**
**Adherence to treatment**				
*N*^b^	577	1,103	1,244	1,117
Mean MPR, % (SD)	87.55 (12.80)	87.73 (14.07)	88.73 (12.73)	89.51 (12.48)
Adherent, *n* (%)^c^	448 (77.6)	878 (79.6)	1,012 (81.3)	938 (84.0)
Non-adherent, *n* (%)^d^	129 (22.4)	225 (20.4)	232 (18.7)	179 (16.0)
**Treatment discontinuation**				
Number of events	334	600	673	583
Patient years	417.97	821.84	937.95	848.48
IR (95% CI)	79.91 (71.57–88.96)	73.01 (67.28–79.09)	71.75 (66.43–77.38)	68.71 (63.25–74.52)
HR (95% CI)^e^	–	0.92 (0.81–1.05)	0.92 (0.80–1.05)	0.88 (0.77–1.01)

a *Reference group for treatment discontinuation*.

b *Patients with data available for calculating MPR*.

c *Adherent: MPR between 80 and 120%*.

d *Non-adherent: MPR <80%*.

e *HR calculated using a Cox proportional hazards model*.

*CI, confidence interval; HR, hazard ratio; IQR, interquartile range; IR, incidence rate; IRR, incidence rate ratio; MPR, medication possession ratio; SD, standard deviation*.

## Discussion

In this cohort analysis of the MarketScan® databases, the proportion of MS patients free of relapse 1 year after treatment initiation with sc IFN β-1a, and the proportion of patients relapse free over the follow-up period, was high among all age groups. However, this may be due to the relatively short duration of follow-up reported in this analysis, for which the median follow-up period was between 6.0 and 7.2 months across the respective age groups.

The ARR and the proportion of patients who were relapse free at 1 year after treatment initiation decreased numerically with increasing age, yet these differences were not statistically significant. This finding is supported by those of previous studies, which have shown that relapses predominantly occur in younger patients and are observed to decrease over time (15), regardless of disease course (16). The lower incidence of relapse observed in the older patient population may be due to a change in the manifestation of MS, in which disease activity is seen to reduce with age while disability progression becomes more of a concern (17).

Our results indicate that fewer MRI scans were performed in patients aged ≥ 51 years compared with younger patients, suggesting a reduced disease activity in the older MS population. However, the decision on how often MRI is performed is based on many factors including, personal preferences of the healthcare provider and/or patient, an individual's safety profile, and whether or not the patient is enrolled in a clinical trial. Recently, there has been a change in guidelines for the follow-up of MS which suggests that MRI scans should be performed every 1–2 years while a patient is treated with DMTs, and that less frequent MRI scans (every 2–3 years) are required in patients with clinically stable MS (18). In the data reported here, more MRI scans may have been conducted in the younger patients possibly due to higher disease activity; however, the results show only a small numerical difference in the proportion of patients experiencing relapse during the 1-year follow-up period. Outpatient visits due to MS increased with age, which may be related to increasing symptoms of disability progression or related concomitant diseases (17).

Treatment adherence describes the successful self-administration of medicine by a patient, and also takes into account the correct usage of treatments (compliance) over time (persistence) (19). In keeping with the results of this study, in which 77.6–84.0% of patients were adherent to treatment over a median follow-up period of 6.0–7.2 months, are the results of an Italian population-based study that also reported high rates of adherence to interferons (>85%) (20). However, others studies have reported lower rates of adherence to IFN β-1a (48.3%) (21) and similarly with IFN β-1b (68.3%) (22). Yet, a systematic literature review of adherence to oral DMTs (dimethyl fumarate, fingolimod and teriflunomide) also found a high 1-year mean MPR of 83.3% (95% CI 74.5–92.1%) based on data from four studies (23). In the present analysis, the proportion of patients considered adherent to treatment increased with age. It is thought that older patients are more adherent to treatment, due to their behavior, whereby they are on treatment for longer, and also as a result of thinking that lower disease activity is due to the effects of sc IFN β-1a treatment rather than it being part of the disease course (24). The rate of adherence seen in the older patient population is similar to that previously reported in a Canadian population-based claims study (25). However, there are other studies that have reported lower proportions of adherent patients 2 years after initiating treatment (26). A further study found that adherence may be related to relapse rates, in which patients who experienced relapses after treatment initiation were more adherent to sc IFN β-1a (27). The rates of adherence reported in the present study are higher than those typically observed in other chronic diseases, such as cardiovascular and psychiatric diseases, in which adherence to long-term therapies is usually 50% (25, 28).

It is worth noting that two methods for assessing patient adherence to prescriptions are commonly used in the literature, namely the proportion of days covered (PDC), and the MPR. The MPR was used in this study as it was possible to use the date of delivery in the MPR calculation, which accurately reflects when the patient obtains their medication, as well as any potential repeat prescriptions. Although MPR may not accurately measure medication adherence compared to PDC, which assesses the extent to which a patient is actually taking the medication as directed, it does assess whether a patient has access to medication. It is also generally accepted that, when examining adherence to a single therapy, MPR and PDC provide nearly identical results (29). For this reason, it was expected that using the MPR would translate to a more accurate representation of adherence than PDC. By using the MPR, adherence can be elevated over 100% when patients obtain their medication in advance of when it is due. Results of this study show that when taking into consideration the SD for the mean MPR, the adherence was found to be slightly elevated over 100% but remained within the adherence category of 80–120% (14).

In this study, treatment discontinuation was observed in a high proportion of patients, and differs to the rates of discontinuation observed by Moccia et al. (20) in which fewer patients (30–45%) discontinued treatment over the 20-month follow-up period (19). A previous real-world cohort analysis using a large population from the MarketScan® databases also reported a discontinuation rate of 36.9% at 1 year (21).

Patients switching treatment were most commonly prescribed dimethyl fumarate, fingolimod, or glatiramer acetate across all ages. A switch from sc IFN β-1a to high-efficacy DMTs may be expected as part of escalating therapy. It is possible that the high number of patients switching to dimethyl fumarate may be due to the study period coinciding with the US approval of the DMT on March 27, 2013.

A limitation of this analysis is that, as a claims database, the diagnosis codes that have been recorded may not always be accurate, and as the study results are dependent on the accuracy of ICD-9-CM or ICD-10 coding in claims files, this may contribute to a certain level of misclassification bias. While it may be possible to identify MS severity in relation to healthcare costs using claims-based algorithms (30), this was not conducted as part of this study. Another possible limitation of this analysis is the lack of detailed clinical data and results for para-clinical examinations provided in claims databases, in which only events accompanying the diagnosis or complicating the disease are encoded. For instance, the clinical staging of MS and the severity of co-morbid medical conditions are not coded for within the MarketScan® databases.

Due to the way in which data were selected for this study, patients may have had claims for MS before the index date, and therefore it is not possible to establish when MS was first diagnosed. Also, because only medically attended events are captured in the MarketScan® databases, it is only possible to identify outcomes requiring healthcare intervention. In the case of co-morbidities or relapses, milder events may not be captured if intervention was not required and therefore the data may be biased toward more serious events. Due to the low number of relapses observed in this study, and because age as a factor cannot be modified by variables, adjusting for confounders via a propensity score would be conceptually difficult, and therefore no adjustments were made for potential confounders. The follow-up period in this study was short and only provides information for the first year after treatment initiation. As such, these results cannot be used to extrapolate to long-term treatment effectiveness and treatment adherence. Additionally, the MarketScan® databases represent working age patients who have healthcare insurance provided by their employers, and therefore represents the active US population. As cases in the MarketScan® databases are employment-based, any interruption to patient employment may have an effect on longitudinal data availability. This study also encompasses the Medicare supplemental data, and thus most of the patients in this study were aged <65 years.

Overall, early initiation of DMTs and treatment adherence are important, however, there are few publications on possible age-related changes in DMT effectiveness in MS. This study, using real-world claims data from 1 year after treatment initiation, demonstrates that the effectiveness of sc IFN β-1a does not appear to reduce as a consequence of age. In older patients, it was observed that the number of relapses decreased with increasing age, whilst the number of outpatient visits related to MS increased with age; possibly as a result of greater disability progression and lower disease activity, which was also reflected in the fewer MRIs performed. Data also showed that adherence increased, and discontinuation decreased with increasing age, similar to previous reports in MS populations. Although relapse activity is still important, progressively, the focus in MS is shifting more toward evaluating disability progression in older patient populations. Further research in this patient population, beyond the median 6.0–7.2-month follow-up reported here, would provide valuable information on how MS can be treated over a patient's lifespan.

## Data Availability Statement

The data analyzed in this study is subject to the following licenses/restrictions: License agreement. Requests to access these datasets should be directed to IBM, https://www.ibm.com/uk-en/products/marketscan-research-databases.

## Ethics Statement

Ethical review and approval was not required for the study on human participants in accordance with the local legislation and institutional requirements. Written informed consent for participation was not required for this study in accordance with the national legislation and the institutional requirements.

## Author Contributions

AA and PV conceptualized and designed the study, and contributed to data analysis and interpretation. EB and MS conceptualized and designed the study, and contributed to data interpretation. KM contributed to data interpretation. All authors were involved in the writing of the manuscript.

## Conflict of Interest

AA was an employee of Merck Healthcare KGaA, Darmstadt, Germany at the time of this study. He is now an employee of Daiichi Sankyo Europe GmbH, Munich, Germany. EB, and MS are employees of Merck Healthcare KGaA, Darmstadt, Germany. KM is an employee of Merck Gesellschaft mbH, Vienna, Austria, an affiliate of Merck KGaA, Darmstadt, Germany. PV has received honoraria or consulting fees from AB Science, Biogen, Celgene (BMS), Imcyse, Merck Healthcare KGaA Darmstadt, Germany, Novartis, Roche, Sanofi-Genzyme, and Teva; and research support from Novartis, Roche, and Sanofi-Genzyme.
