# Supplementary material for: Effectiveness, Healthcare Resource Utilization and Adherence to Subcutaneous Interferon Beta-1a According to Age in Patients With Multiple Sclerosis: A Cohort Study Using a US Claims Database
Source: Front Neurol. 2021 Jul 22;12:676585. doi: 10.3389/fneur.2021.676585 (PMC8351462; doi:10.3389/fneur.2021.676585)
Supplement: Supplementary file 1 [file Table_1.docx]

**Supplementary Appendix**

**Supplementary Methods**

*Relapse algorithm*

Relapse episodes will be identified based on a published claims-based algorithm (Marriott, 2018). The algorithm has a sensitivity of 70%, specificity of 100%. This claims-based algorithm correctly classified 100% of patients with relapses (positive predictive value) and 96% of patients without relapses (negative predictive value; kappa 0.8). A patient will then be considered as having experienced a moderate/severe relapse if he/she has oral prednisone prescriptions >50 mg/day (or equivalent corticosteriods such as betamethasone >8mg/day, dexamethasone >8mg/day, methylprednisolone >40mg/day, prednisolone >50 mg/day) for 3–60 days or same day hospital or ER assessment codes with MS as the most responsible diagnosis. As patients may have had multiple clinical encounters during one relapse, all qualified hospitalizations or outpatient visits that occurred within 30 days of each other will be considered as one relapse episode.

Reference: Marriott JJ, Chen H, Fransoo R, Marrie RA. Validation of an algorithm to detect severe MS relapses in administrative health databases. Mult Scler Relat Disord. 2018;19:134-139. doi: 10.1016/j.msard.2017.11.022.

**Supplementary Tables**

Supplementary Table S1. Frequency of common co-morbidities and co-medications at 1 year after initiation of sc IFN β-1a treatment

| **% (95% CI)** | **Age group, years** | | | |
| --- | --- | --- | --- | --- |
|  | **18–30**  **(N=773)** | **31–40**  **(N=1468)** | **41–50**  **(N=1630)** | **≥51**  **(N=1469)** |
| **Co-morbidities** | | | | |
| Alcohol use disorder | 0.5 (0.2–1.3) | 0.5 (0.3–1.1) | 0.5 (0.3–1.0) | 0.1 (0.0–0.4) |
| COPD | 0.4 (0.1–1.1) | 0.3 (0.2–0.8) | 0.7 (0.4–1.3) | 2.3 (1.7–3.2) |
| Diabetes mellitus | 1.2 (0.6–2.2) | 2.4 (1.7–3.3) | 6.3 (5.2–7.5) | 10.5 (9.0–12.2) |
| Autoimmune disorders | 3.2 (2.2–4.7) | 4.8 (3.8–6.0) | 6.8 (5.7–8.1) | 6.3 (5.2–7.7) |
| Depression | 8.9 (7.1–11.1) | 9.3 (7.9–10.9) | 11.6 (10.1–13.2) | 11.4 (9.9–13.2) |
| Dementia | 0.1 (0.0–0.7) | 0.4 (0.2–0.9) | 0.9 (0.5–1.4) | 2.3 (1.6–3.1) |
| Hypertension | 3.2 (2.2–4.7) | 8.2 (6.9–9.8) | 16.8 (15.0–18.6) | 29.3 (27.0–31.7) |
| **Co-medications** | | | | |
| Fatigue | 5.4 (4.0–7.3) | 6.7 (5.5–8.1) | 10.6 (9.2–12.2) | 10.4 (8.9–12.0) |
| Bladder dysfunction | 12.2 (10.0–14.7) | 14.0 (12.3–15.8) | 21.3 (19.4–23.3) | 28.9 (26.6–31.2) |
| Urinary tract infection | 6.9 (5.3–8.9) | 5.5 (4.4–6.7) | 7.4 (6.3–8.8) | 9.3 (7.9–10.9) |
| Bowel problems | 0.4 (0.1–1.1) | 0.9 (0.5–1.5) | 2.0 (1.4–2.8) | 3.7 (2.9–4.8) |
| Spasticity | 20.8 (18.1–23.8) | 24.5 (22.4–26.8) | 30.7 (28.5–33.0) | 35.8 (33.4–38.3) |
| Walking impairment | 1.0 (0.5–2.0) | 1.2 (0.7–1.9) | 4.0 (3.1–5.1) | 6.9 (5.7–8.3) |
| Contracture at joints | 0.1 (0.0–0.7) | 0.5 (0.3–1.1) | 1.0 (0.5–2.0) | 1.1 (0.7–1.8) |
| Pain | 21.2 (18.5–24.4) | 25.8 (23.6–28.1) | 28.8 (26.7–31.1) | 31.0 (28.7–33.4) |
| Emotionalism | 17.9 (15.3–20.7) | 23.0 (20.9–25.3) | 23.8 (21.8–25.9) | 26.6 (24.4–28.9) |
| Depression | 28.7 (25.6–32.0) | 37.7 (35.5–40.2) | 40.0 (37.7–42.4) | 44.7 (42.2–47.3) |

CI, confidence interval; COPD, chronic obstructive pulmonary disorder; sc IFN β-1a; subcutaneous interferon beta-1a.

Supplementary Table S2. Proportion of patients who switched treatment, and the second-line treatment received

|  | **Age group, years** | | | |
| --- | --- | --- | --- | --- |
|  | **18–30**  **(N=773)** | **31–40**  **(N=1468)** | **41–50**  **(N=1630)** | **≥51**  **(N=1469)** |
| Second-line treatment, n (%) |  |  |  |  |
| Aubagio® (teriflunomide) | 10 (4.3) | 30 (6.7) | 59 (10.6) | 38 (8.0) |
| Avonex® (interferon β-1a) | 13 (5.6) | 29 (10.8) | 57 (10.3) | 86 (18.0) |
| Betaferon® (interferon β-1b) | 4 (1.7) | 19 (4.3) | 18 (3.2) | 16 (3.3) |
| CellCept® (mycophenolate mofetil) | 0 (0) | 0 (0) | 1 (0.2) | 1 (0.2) |
| Copaxone® (glatiramer acetate) | 53 (22.8) | 103 (23.2) | 124 (22.3) | 102 (21.3) |
| Cyclophosphamide | 0 (0) | 0 (0) | 0 (0) | 1 (0.2) |
| Extavia® (interferon β-1b) | 0 (0) | 1 (0.2) | 0 (0) | 1 (0.2) |
| Gilenya® (fingolimod) | 68 (29.2) | 101 (22.7) | 93 (16.7) | 61 (12.7) |
| Lemtrada® (alemtuzumab) | 0 (0) | 1 (0.2) | 0 (0) | 0 (0) |
| Methotrexate sodium | 1 (0.4) | 1 (0.2) | 6 (1.1) | 6 (1.3) |
| Mycophenolate mofetil | 1 (0.4) | 0 (0) | 4 (0.7) | 3 (0.6) |
| Ocrevus® (ocrelizumab) | 0 (0) | 3 (0.7) | 0 (0) | 1 (0.2) |
| Plegridy® (pegylated interferon β-1a) | 3 (1.3) | 10 (1.1) | 11 (1.6) | 12 (2.5) |
| Tecfidera® (dimethyl fumarate) | 72 (30.9) | 130 (29.2) | 168 (30.2) | 139 (29.0) |
| Tysabri® (natalizumab) | 6 (2.6) | 14 (3.2) | 9 (1.6) | 8 (1.7) |
| Zinbryta® (daclizumab) | 0 (0) | 1 (0.2) | 1 (0.2) | 1 (0.2) |
| Total number who switched treatment | 233 (30.1) | 445 (30.3) | 556 (34.1) | 479 (32.6) |

Supplementary Table S3. Sensitivity analyses of patients relapse-free at 1 year after initiation of sc IFN β-1a treatment

|  | **Age group, years** | | | |
| --- | --- | --- | --- | --- |
| Relapse-free at 1 year | **18–30^a^**  **(N=773)** | **31–40**  **(N=1468)** | **41–50**  **(N=1630)** | **≥51**  **(N=1469)** |
| Gap window of 90 days following discontinuation^b^ | | | | |
| Proportion of patients (95% CI) | 92.10  (90.11–94.10) | 92.65  (91.22–94.08) | 94.10  (92.86–95.33) | 94.31  (93.05–95.57) |
| Unadjusted SHR (95% CI) | - | 0.89  (0.64–1.24) | 0.69  (0.49–0.96) | 0.68  (0.48–0.96) |
| *p* value | - | 0.490 | 0.030 | 0.029 |
| Baseline of 12 months^c^ | | | | |
| Proportion of patients 95% CI) | 94.79  (92.79–96.79) | 94.23  (92.67–95.78) | 95.44  (94.15–96.72) | 95.08  (93.73–96.43) |
| Unadjusted SHR (95% CI) | - | 1.05  (0.65–1.69) | 0.80  (0.49–1.30) | 0.89  (0.55–1.45) |
| *p* value | - | 0.85 | 0.36 | 0.65 |

^a^Reference group; ^b^Sensitivity analysis considering a gap window of 90 days instead of no gap following discontinuation to account for any of the relapse events; ^c^Sensitivity analysis using a patient history prior of at least 12 months (vs ≥6 months) prior to the index date.

CI, confidence interval; sc IFN β-1a, subcutaneous interferon beta-1a; SHR, subdistribution hazard ratio estimates.
